# A single N342D substitution in Influenza B Virus NA protein determines viral pathogenicity in mice

**DOI:** 10.1080/22221751.2020.1806005

**Published:** 2020-08-21

**Authors:** Lijuan Zhou, Zhaomin Feng, Jia Liu, Yongkun Chen, Lei Yang, Suli Liu, Xiyan Li, Rongbao Gao, Wenfei Zhu, Dayan Wang, Yuelong Shu

**Affiliations:** a School of Public Health (Shenzhen), Sun Yat-sen University, Guangdong, People’s Republic of China; b National Institute for Viral Disease Control and Prevention, Chinese Center for Disease Control and Prevention, Beijing, People’s Republic of China

**Keywords:** Influenza B virus (IBV), neuraminidase, pathogenicity, molecular marker

## Abstract

Influenza B virus (IBV) is one of the most important human respiratory viruses: it causes approximately one-third of the global influenza-related disease burden each year. However, compared with the several pathogenicity-related molecular markers that have been identified for influenza A virus (IAV), little is known about potential IBV pathogenicity-related markers. Here, although the IBV strain B/Anhui-Tunxi/1528/2014 (AH1528/14) exhibited a more efficient replication ability *in vitro* and higher pathogenicity *in vivo* compared with IBV strain B/Anhui-Baohe/127/2015 (AH127/15), only three amino acids differences (HA_A390E_, NA_N342D_ and PB1_V212I_) were observed among their full genomes. The contributions of each amino acid difference to the virus pathogenicity were further investigated. Compared with the wild type IBV virus rAH127, the recombinant virus harbouring a single substitution of HA_A390E_ had a similar phenotype, whereas the recombinant virus harbouring PB1_V212I_ replicated to a moderately higher titre in both MDCK cells and in mice. Notably, the virus harbouring NA_N342D_ showed significantly better growth properties in MDCK cells and higher fatality rates in mice. In addition, the presence of NA_N342D_ dramatically enhanced the viral neuraminidase activity. In conclusion, our study identified a novel IBV molecular marker, NA_N342D_, that could significantly increase the virulence of IBV in mice.

## Introduction

Influenza B virus (IBV) and influenza A virus (IAV) (H1N1 and H3N2 subtypes) are currently co-circulating widely in human populations, leading to annual epidemics with significant morbidity and mortality. IBV infections account for 20%-30% of total seasonal influenza cases [1]. In some influenza seasons, the IBV detection rate and disease burden were even higher than those of IAV. For example, from 2002 to 2003, the mortality rate of IBV infections was as high as 78% in the United States [2]. During the 2017/2018 season, 60% and 54.3% of laboratory-confirmed influenza infections were associated with IBV in Europe[3] and China [4], respectively. Thus, in addition to IAVs, IBVs also pose a great threaten to public health.

The key genetic determinants that contribute to viral adaptation and pathogenicity in IAV have been broadly investigated in numerous studies. A substitution from E to K at position 627 in the PB2 protein significantly increased the pathogenicity of avian influenza viruses (AIVs) in mice [5–7]. A D701N mutation in the PB2 protein led to effective viral replication and enhanced the pathogenicity of both AIVs and swine influenza virus in mammals [8,9]. Other molecular markers involved with IAV mammalian adaptation have been identified as well, including PB2_Q591K_ [10,11], PB2_A588V_ [12], and NPQ35_7K_ [13].

In contrast, the molecular determinants of IBV pathogenicity are relatively poorly understood. Currently, the identifications of IBV pathogenic determinants were exclusively performed by serially lung-lung passaged in mouse model. Mccullers et al. [14] reported that after serial passages of an initially avirulent IBV in mice, a selected variant that was lethal to mice was appeared. Sequencing and site-directed mutagenesis revealed that M1_N221S_ was responsible for the acquisition of virulence and lethality. Bae et al. [15] reported that compared with wild-type viruses, two mouse-adapted IBVs generated by passages through mice both harboured the mutation PA_K338R_. Further study showed that PA_K338R_ alone could increase the pathogenicity of IBVs in mice. Another study performed by Kim et al. [16] identified multiple adaptive mutations of IBV, including HA_D424G_, M1_N240T_, M2_N14S_, NP_K294N_, and PA_V609I_. The combination of these mutations significantly enhanced viral replication in mice and viral transmission in ferrets.

IBVs were classified into two antigenically-distinct lineages in the 1970s, including B/Victoria and B/Yamagata [17]. Both lineages have co-circulated during each influenza season since at least 2001, and they are usually alternately dominant [18,19]. In mainland China, B/Yamagata lineage viruses showed higher levels of epidemic activity compared with B/Victoria lineage viruses and led to three major epidemics during the 2013/2014, 2014/2015 and 2017/2018 seasons [4,20]. Therefore, in this study, we evaluated the evolution and biological characteristics of B/Yamagata lineage viruses, and further investigated their pathogenicity determinants.

## Materials and methods

### Ethics statement

All mouse experiments and the related animal care and handling, including mice housing, feeding, virus infection and sample collection were approved by the Ethics Committee of the National Institute for Viral Disease Control and Prevention, China CDC (20190212004). All experiments using live viruses were performed in biosafety level 2 (BSL2) laboratories.

### Phylogenetic analysis

The whole-genome sequences of IBV (Yamagata lineage) isolated in mainland China during 2014–2018 (546 HA, 502 NA, 30 PB2, 23 PB1, 33 PA, 32 NP, 387 M, and 33 NS sequences) were downloaded from the Global Initiative on Sharing All Influenza Data (GISAID). Sequences were aligned using MEGA 7.0 software [21]. Phylogenetic trees were generated by applying the maximum likelihood method with the general time-reversible (GTR) model. The robustness of the ML topology was determined using 1,000 bootstrap replicates.

### Cells and viruses

Madin–Darby canine kidney cells (MDCK) and human embryonic kidney (293T) cells were maintained in Dulbecco’s Modified Eagle’s Medium (DMEM, Invitrogen, Carlsbad, CA, USA) supplemented with 10% foetal bovine serum (FBS, Invitrogen), HEPES (10 mM, Invitrogen), penicillin (100 units/mL, Invitrogen), and streptomycin (100 µg/mL, Invitrogen). The wild type IBV strains, B/Shanghai-Songjiang/1101/2017, B/Neimenggu-Dongsheng/11/2018, B/Sichuan-Qingyang/52/2017, B/Anhui-Tunxi/1528/2014, B/Anhui-Baohe/127/2015 and B/Shanghai-Minxing/1485/2015 were propagated in MDCK cells. After being incubated at 33°C for 72 h, the viruses were harvested and stored at −80°C until use. Virus titrations were determined in MDCK cells. All viruses were sequenced before their use in experiments.

### Plasmid construction and recombinant virus rescue

The eight gene segments of IBV were each cloned into a bidirectional pHW2000 vector. The protocol and primers used for the construction of these eight plasmids were based on the reference strategy [22]. Mutations in the HA, PB1, and NA segments were generated by PCR amplification of the recombinant pHW2000-HA/PB1/NA plasmids with primers possessing the desired mutations. Recombinant IBVs were generated by co-transfection of 293T/MDCK cells with the eight reverse-genetics plasmids carrying the cDNA of each gene segment for 24 h as previously described [23,24]. The culture supernatants were collected and the viruses were propagated in MDCK cells at 33°C for 72 h. All rescued viruses were sequenced completely to ensure they did not contain any unwanted mutations.

### Growth kinetics

MDCK cells were infected with viruses at a multiplicity of infection (MOI) of 0.001. After 1h of incubation at 37°C, the viral inoculums were removed and the cells were washed twice with PBS. Infection medium containing TPCK-trypsin (1 µg/mL) was then added, and the cells were incubated at 37°C with 5% CO_2_. The cell supernatant was collected at 0, 24, 48, 72 and 96 h post-infection (h.p.i.). The viral titre was determined by measuring the TCID_50_ in MDCK cells.

### Mous experiments

Eight-week-old specific-pathogen-free female C57BL/6 mice [SPF (Beijing) Biotechnology Co.,Ltd.] were used in all experiments. Under the anaesthesia induced by pentobarbital sodium, five mice per group were intranasally infected with PBS, 10^1^, 10^2^, 10^3^, 10^4^, 10^5^, 10^6^, or 10^7^ TCID_50_ (50 µL) of virus. The bodyweight and survival of mice were monitored daily for 14 days, and mice with a 25% weight loss were euthanized for humane reasons. For determination of the 50% mouse infectious dose (MID_50_), at 14 days post infection (d.p.i.), serum samples were collected from the surviving mice. After being treated with receptor-destroying enzyme (Denka Seiken) for 18 h at 37°C and being inactivated at 56°C for 30 min, the hemagglutinin inhibiting (HI) antibody titres of the serum samples were determined with 0.5% (vol/vol) turkey erythrocytes, HI titres ≥40 were considered to be antibody positive. The calculation of MID_50_ was performed using the Karber method. For viral replication and pathogenicity assessments, six mice per group were inoculated intranasally with 10^7^ TCID_50_ (50 µL) of viruses, three of which were euthanized at 1 d.p.i., and three of which were euthanized at 4d.p.i., three mice per group were inoculated intranasally with PBS (50 µL) and euthanized 1 d later. The lung and snout were harvested from these mice, and their viral titre was determined in MDCK cells by applying the TCID_50_ method. The right lung lobe from each mouse was preserved in 10% formalin, embedded in paraffin and cut into 4-µm-thick sections. One section from each sample was stained with haematoxylin and eosin. The 50% median lethal dose (MLD_50_) was determined as described previously using five mice infected with each virus dilution and the Karber calculation method [25].

### Cytokine and chemokine detections

Total RNA was extracted from murine lung tissue by using the QIAamp Viral RNA Mini Kit (Qiagen) in accordance with the manufacturer’s instructions. Total RNA was reverse transcribed into cDNA by using a PrimeScript™ RT reagent Kit with gDNA Eraser (Perfect Real Time, Takara), and real-time PCR was performed with Roche light cycler96 instrument using SYBR Premix Ex Taq (Takara). The primers for β-actin, IL-6, CCL5 [26], IL-8 [27], IL-4, IL-10 [28] and IFN-α [29] were designed as previously described. The target genes expression levels were normalized to β-actin mRNA and calculated by the relative quantitation (change in cycle threshold2^−ΔΔt^) method.

### Polymerase activity

The pHW2000 plasmids encoding the PB2, PB1, PA and NP genes were co-transfected with the firefly RNA luciferase reporter plasmid and the Renilla luciferase-expressing plasmid pRL-TK using SuperFect Transfection Reagent in 293T cells. The firefly luciferase and Renilla activity were measured with the dual-luciferase reporter assay at 24 h post-transfection.

### Representation of three-dimensional protein structures and neuraminidase activity assay

Three-dimensional structures of neuraminidase proteins (PDB: 1INF) were obtained from the protein data bank (www.rscb.org) and displayed using the PyMOL software (www.pymol.org).

NA-Fluor Influenza Neuraminidase Assay kit (Applied Biosystem) was used to measure the neuraminidase activity. Briefly, the viruses were serially diluted two-fold from 1×10^6^ TCID_50_/50 µL as described [30]. Then, 50 µL of each virus was mixed with 50 µL of 200 µM NA-Fluor substrate working solution and incubated at 37°C for 1 h. The reaction was then terminated by adding 100µL of NA-Fluor stop solution, and the fluorescence was measured using excitation and emission wavelengths of 365 and 450 nm, respectively. To determine the viral particle number, total RNA was extracted from 50 µL of serially diluted viruses by using Mag Max Core Nucleic Acid Purification Kit (Applied Biosystem), and the copy number of NS1gene were absolute Quantificated by performing digital PCR.

### Statistical analysis

All determinations were performed at least three times. The Student’s *t*-test was used for statistical analysis, and differences were considered significant at *p­*-values of <0.05.

## Results

### Phylogenetic analysis of IBVs

**Figure 1.**
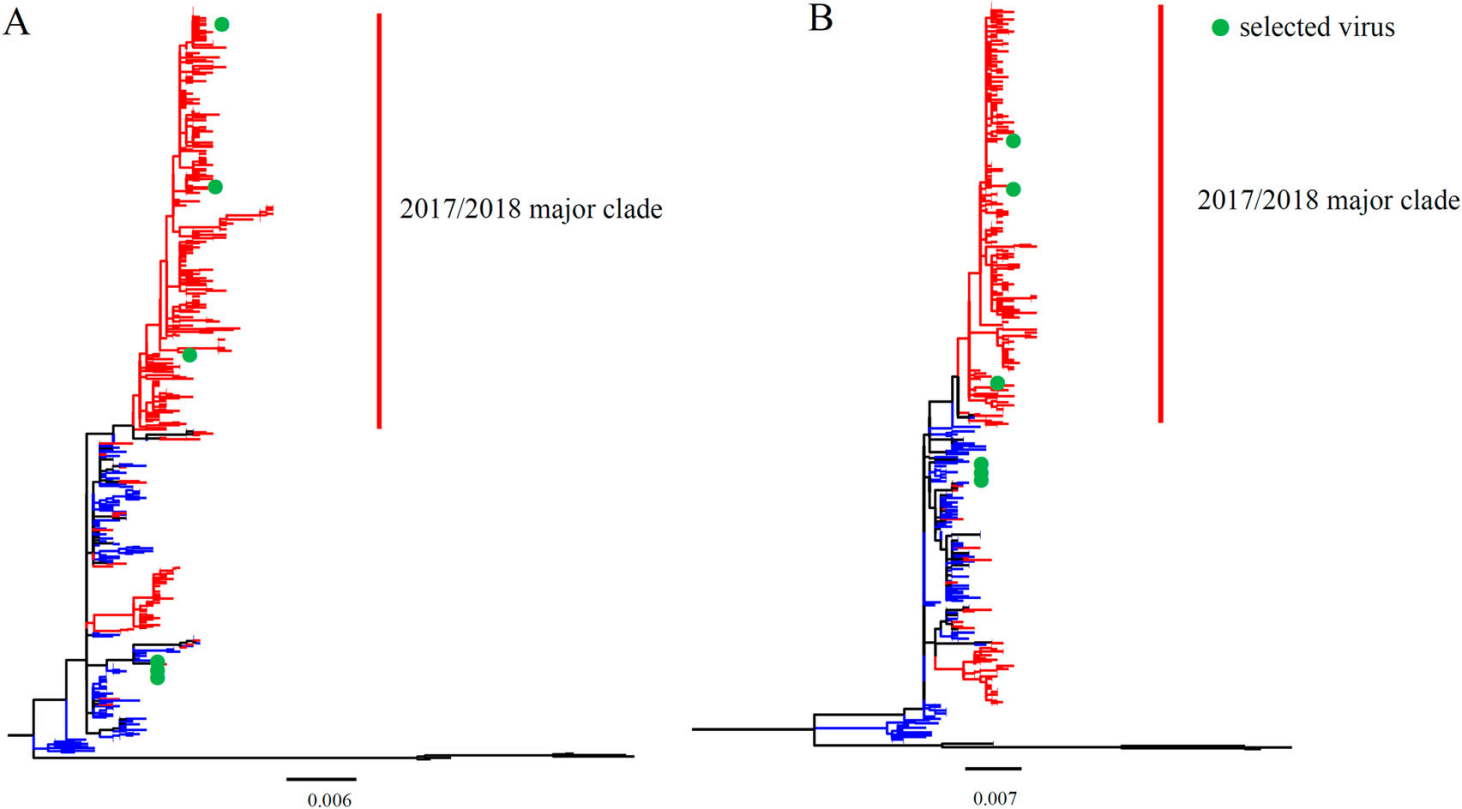
Phylogenetic analysis of the IBV HA and NA genes. The unrooted trees were constructed using the neighbour-joining method with the  maximum composite likelihood model and MEGA version 7 with 1000 bootstrap replicates. (A–B) Complete HA (A) or NA (B) gene nucleotide sequences of IBV isolated during 2014–2018 from human in mainland China were download from GISAID. The red lines indicate viruses from the 2017/2018 year, the blue lines indicate viruses belonged to pre-2017/2018 year, and the green points indicate the viruses used in the present research.

To assess the evolution of HA and NA genes, we phylogenetically analysed the sequences of B/Yamagata lineage viruses isolated in mainland China during 2014–2018. Phylogenetic trees of HA and NA each revealed that viruses isolated during 2017/2018 formed a major and a minor clade (Figure 1). In the major clades of HA and NA, there were 934 (total: 1092) and 257(total: 335) strains, respectively. Although the number of sequences available for each of the six internal IBV genes was limited, similar evolutionary patterns were shown in the phylogenetic trees of all the six internal genes (Figure S1).

### *In vitro* and *in vivo* IBV characterizations

To compare the pathogenicity of IBVs between the “2017/2018 major clade” and pre-2017/2018 year viruses, six clinical IBVs were selected for further study based on the phylogenetic trees and the availability of whole genome-wide sequences and strain resources in our laboratory. B/Shanghai-Songjiang/1101/2017 (SH1101/17), B/Neimenggu-Dongsheng/11/2018 (NMG11/18), and B/Sichuan-Qingyang/52/2017 (SC52/17) were selected from 2017/2018 major clade. B/Anhui-Tunxi/1528/2014 (AH1528/14), B/Anhui-Baohe/127/2015 (AH127/15), and B/Shanghai-Minxing/1485/2015 (SH1485/15) were selected from pre-2017/2018 year viruses.

**Figure 2.**
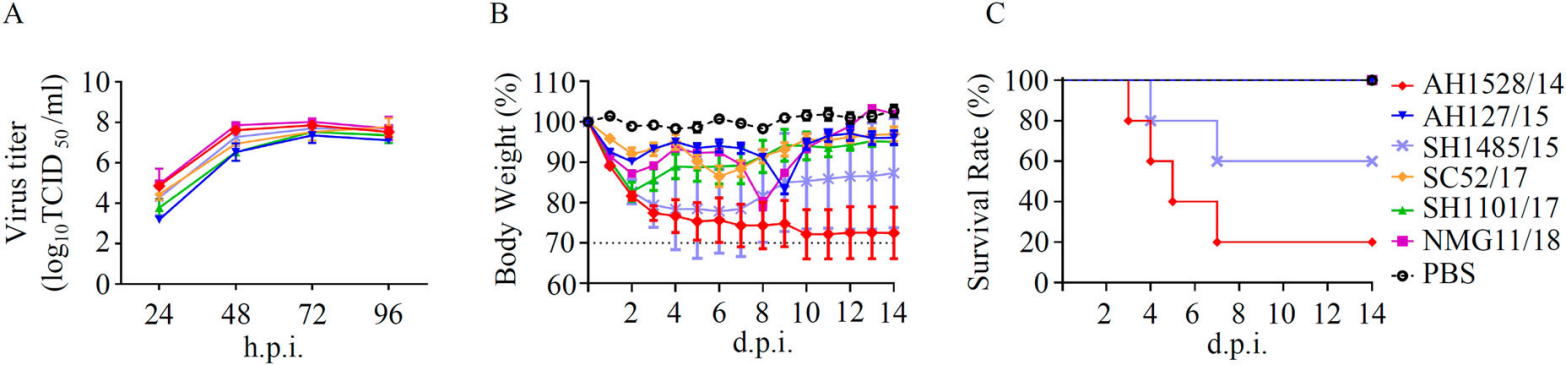
*In vitro* and *in vivo* characterizations of IBV. (A) Growth kinetics of different IBVs in MDCK cells at 37°C. MDCK cells were infected with AH1528/14, AH127/15, SC52/17, NMG11/18, SH1101/17, or SH1485/15 at an MOI of 0.001. The supernatant of the infected cells was harvested at the indicated times. The viral titres were determined as the TCID_50_ in MDCK cells. The presented results are the means of three independent experiments, and the error bars show the standard errors of the mean (SEM). (B–C) Bodyweight loss (B) and survival rate (C) of mice infected with various IBV strains. Groups of mice (*n* = 5) were intranasally infected with 10^7^ TCID_50_ of each virus or PBS. The bodyweight loss and the survival of the animals were monitored through 14 d.p.i.

The growth-kinetics properties of the selected viruses in MDCK cells were compared. As shown in Figure 2(A), AH127/15 showed the lowest viral titres at all indicated timepoints among the tested viruses. AH1528/14 and NMG11/18 replicated equally efficient and showed the highest viral titres. Compared with AH127/15, the titres of these two viruses were 31.6- and 10-fold higher at 24 and 48 h.p.i., respectively (24 h.p.i. *p *<* *0.0001; 48 h.p.i. *p *<* *0.05). These results indicate that while AH127/15 showed the lowest viral titres in mammalian cells, growth properties of AH1528/14 and NMG1 were higher than those of the other tested viruses.

To determine the pathogenicity of these IBVs in mice, we intranasally infected 8-week-old female C57B/L6 mice with 10^7^ TCID_50_ of each virus. The survival and bodyweight loss the animals were monitored until 14 d.p.i.. Data showed that viruses in 2017/2018 major clade did not induce any clinical symptoms, whereas, the viruses from pre-2017/2018 year caused different pathogenicity phenotypes. AH1528/14 and SH1485/15 caused symptoms of illness and obvious bodyweight loss from 1 d.p.i. in mice, and led to fatality rates of 80% and 40% respectively. In contrast, mice infected with AH127/15 all survived, with only a small loss in bodyweight loss (Figure 2(B,C)). In summary, corresponding to the virus growth properties in MDCK cells, AH1528/14 caused the highest fatality rates (80%) in mice, and AH127/15 exhibited low pathogenicity in mice.

### Infectivity, virulence, and replication of AH1528/14 and AH127/15 in mice

**Figure 3.**
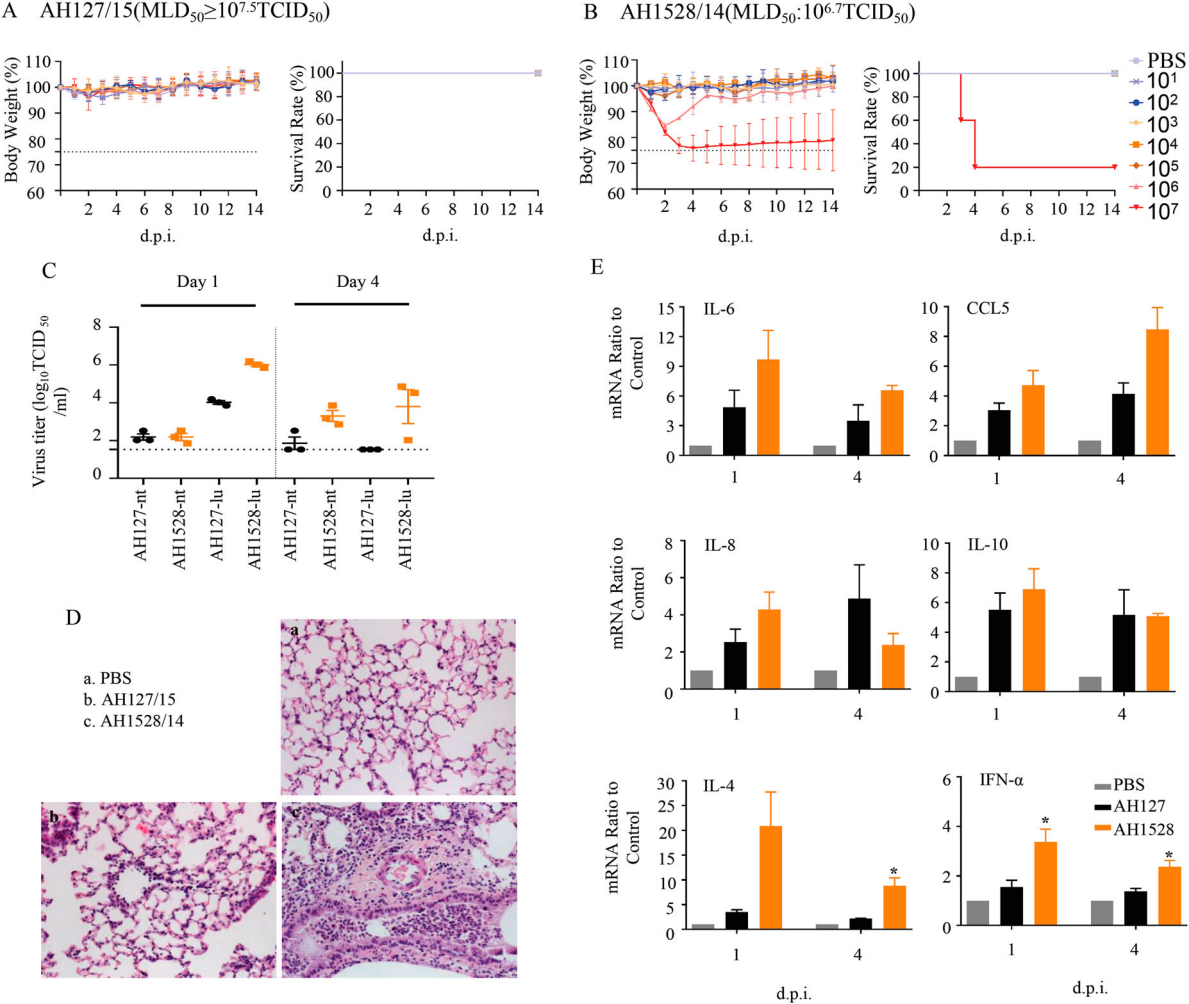
Infectivity, virulence, and replication of AH1528/14 and AH127/15 in mice. (A–B) Five mice per group were inoculated intranasally with PBS (control) or with 10^1–^10^7^ TCID_50_ of AH127/15 (A) or AH1528/14 (B). Bodyweight changes and survival of the mice were monitored daily. (C)Mice were infected with 10^7^ TCID_50_ of wildtype AH127/15 or AH1528/14, 1 and 4 days later three mice per group were euthanized. The viral titres in tissues were determined by performing TCID_50_ assays. (D) The histopathological lesions in the lungs of mice infected with PBS (a), AH127/15 (b) or AH1528/14 (c). Scale bars, 100 µm. (E) The mRNA levels of cytokines and chemokines in the lungs were determined by performing real-time PCR. Error bars for body weight show the standard errors of the mean (SEM). The statistical significance compared with AH127/15-infected mice was determined by a *t*-test, *indicates *p *< 0.05.

To further investigate the pathogenicity of the two representative IBV viruses (AH1528/14 and AH127/15) in mice, we evaluated their infectivity, virulence, replication, tissue tropism, and cytokine/chemokine expression levels after host inoculation. As expected, the MLD_50_ value of AH1528/14 (10^6.7^ TCID_50_) was less than that of AH127/15 (≥10^7.5^ TCID_50_) (Table 1, Figure 3(A,B)). According to the HI antibody test results, the MID_50_ value of AH1528/14 was 10^0.7^ TCID_50_, which is almost 100-fold lower than that of AH127/15 (10^3.3^ TCID_50_) (Table 1, Supplementary Table 1). These data demonstrated the higher virulence of the AH1528/14 virus compared with AH127/15 virus in mice.

**Table 1.** MLD_50_ and MID_50_ of the wild type and recombinant viruses.

Viruses	^a^MLD_50_（TCID_50_）	^b^MID_50_(log TCID_50_)
AH1528/14	10^6^.^7^	0.7
AH127/15	≥10^7^.^5^	3.3
rAH127	≥10^7^.^5^	2.5
rAH127/NA_N342D_	10^6^.^5^	1.9
rAH127/PB1_V212I_	10^6^.^9^	2.3
rAH127/NA_N342D_+PB1_V212I_	10^6^.^5^	2.7
rAH127/HA_A390E_	≥10^7^.^5^	1.7
rAH127/HA_A390E_+NA_N342D_	10^6^.^7^	1.7
rAH127/HA + PB1_V212I_	10^6^.^9^	1.9
rAH127/HA_A390E_+NA_N342D_+PB1_V212I_	10^6^.^5^	0.7

^a^MLD_50_,50％ mouse lethal dose, and was determined using the Karber Method.

^b^MID_50_, 50％ mouse infection dose, and was determined using the Karber Method.

Brain, nasal turbinate, lung, heart, spleen, kidney, liver, and intestines were collected from infected mice. Viruses were only detected in only the respiratory tissues, i.e. the lung and nasal turbinate. Although AH127/15 virus exhibited a nasal turbinate viral titre (2.2 log_10_ TCID_50_/mL) similar to that of AH1528/14 at 1 d.p.i., the lung viral titre of AH1528/14(6.02 log_10_ TCID_50_/mL) was 100-fold higher than that of AH127/15 (4.02 log_10_ TCID_50_/mL) in the lung (*p* < 0.0001). At 4 d.p.i, viruses could be detected only in the upper airway of one mouse inoculated with the AH127/15, and no virus was detected in the lung. In contrast, the AH1528/14 replicated efficiently in both the nasal turbinate (3.3 log_10_ TCID_50_/mL, *p < *0.05) and lung (3.8 log_10_ TCID_50_/mL, *p* < 0.05) (Figure 3(C)). These data suggest that AH1528/14 replicated more efficiently in the upper airway and lungs of mice compared with AH127/15. In addition, more severe bronchopneumonia, more severe exfoliation and degeneration of bronchial epithelial cells, and more inflammatory cell infiltration were observed in the lungs of AH1528/14-infected mouse group compared with AH127/15-infected mouse group (Figure 3(D)). Influenza virus infection triggers inflammatory responses, and the induced cytokines and chemokines such as IL-6, IL-8, IL-10, CCL5, and IFN-α may contribute to disease severity [31,32]. A detailed analysis of the chemokines and cytokine expression changes demonstrated that both groups of animals showed activation of several inflammatory chemokine and cytokine genes. Levels of IFN-α, IL-4, IL-6 and CCL5 expression in the lungs of AH1528/14-infected mice were higher than those of the AH127/15-infected group at both 1 and 4 d.p.i. (Figure 3(E)). Taken together, these results indicated that AH1528/14 has higher virulence and pathogenicity compared with AH127/15.

### NA_N342D_ increased the in vitro viral pathogenicity

**Figure 4.**
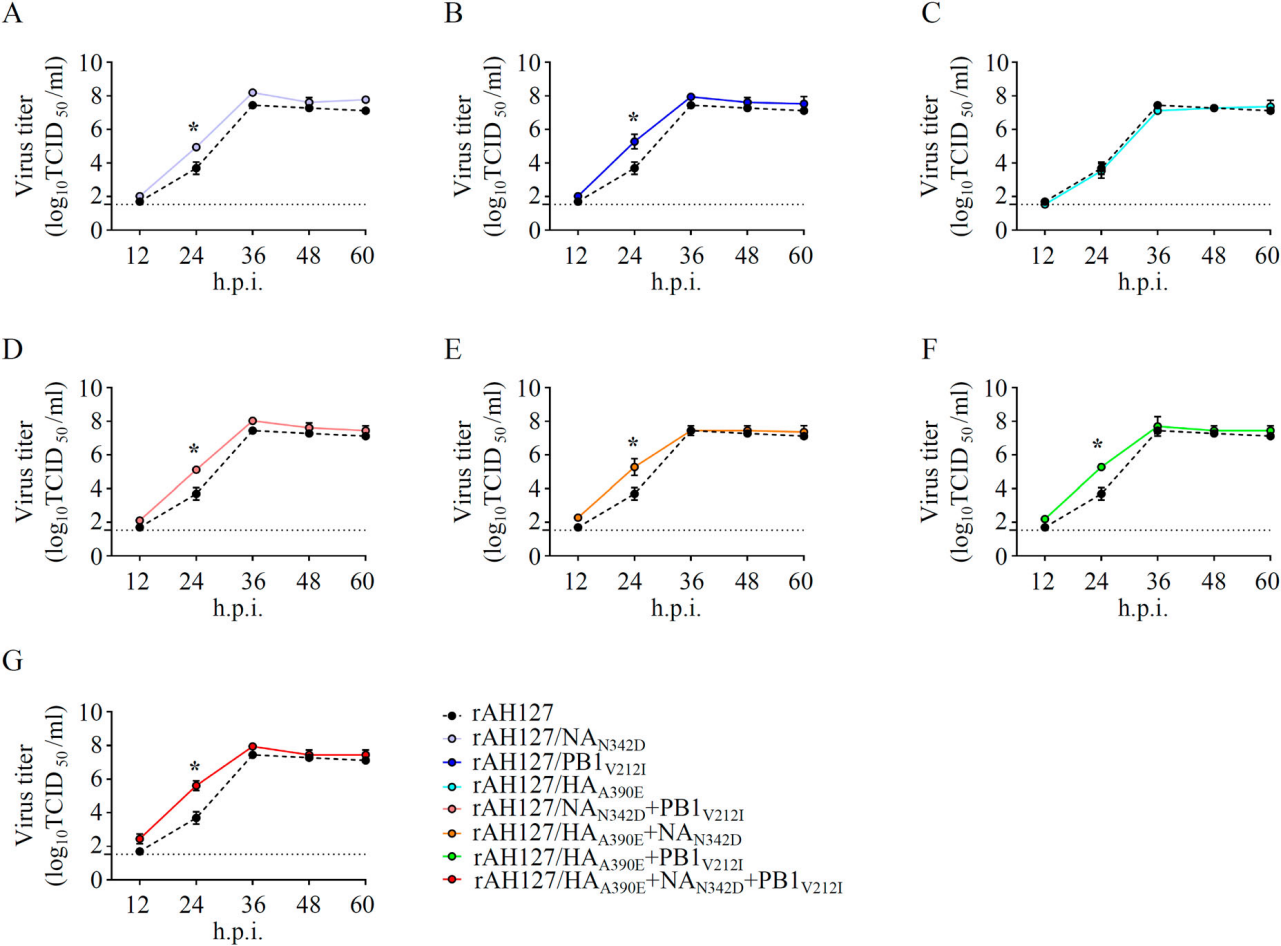
*In vitro* growth kinetics of the recombinant IBVs. (A–G) MDCK cells were infected with rAH127, rAH127/NA_N342D_ (A), rAH127/PB1_V212I_ (B), rAH127/HA_A390E_ (C), rAH127/NA_N342D_+PB1_V212I_ (D), rAH127/HA_A390E_+NA_N342D_ (E), rAH127/HA_A390E_+PB1_V212I_ (F), or rAH127/HA_A390E_+NA_N342D_+PB1_V212I_ (G) at an MOI of 0.001. Culture supernatants were harvested at 0, 12, 24, 36, 48, and 60 h.p.i. and the titres were determined by performing TCID_50_ assays in MDCK cells. The presented results are the means of three independent experiments, and the error bars show the standard errors of the mean (SEM).

To identify the key amino acid substitution that contributed to the relatively high pathogenicity of AH1528/14 in mice, we first aligned the whole genome sequences of AH1528/14 and AH127/15. The result showed that the AH1528/14 differs from AH127/15 by only three amino acids (NA_N342D_, PB1_V212I,_ and HA_A390E_; Table 2). To further explore the impacts of these three substitutions on IBV pathogenicity, we used reverse genetics to generated the mutant viruses with an AH127/15 backbone harbouring a single or multiple combined substitutions: the wild type rAH127, rAH127/NA_N342D_, rAH127/PB1_V212I_, rAH127/NA_N342D_+PB1_V212I_, rAH127/HA_A390E_, rAH127/HA_A390E_+NA_N342D_, rAH127/HA_A390E_+PB1_V212I_ and rAH127/HA_A390E_+NA_N342D_+PB1_V212I_. As shown in Figure 4, rAH127/HA_A390E_ replicated similarly to rAH127 (Figure 4(C)), whereas the other two recombinant viruses with single mutations (NA_N342D_ or PB1_V212I_) exhibited relatively enhanced replication at 24 h.p.i. (*p* < 0.05) (Figure 4(A,B)). All three dual-mutants (rAH127/NA_N342D_+PB1_V212I_, rAH127/HA_A390E_+NA_N342D_, and rAH127/HA_A390E_+PB1_V212I_) also replicated better compared with rAH127 at 24 h.p.i. (*p* < *0.05*) (Figure 4(D–F)). Additionally, the triple mutant rAH127/HA_A390E_+NA_N342D_+PB1_V212I_ replicated to significantly higher titres than rAH127 at 24 h.p.i. (*p* < *0.05*) (Figure 4(G)). These results indicate that the NA_N342D_ and/or PB1_V212I_ mutations could affect the viral replication of IBV *in vitro*.

**Table 2.** Amino acid substitutions between AH1528/14 and AH127/15

Substitutions	Amino acid	
	AH1528/14	AH127/15
PB1-212	I	V
NA-342	D	N
HA-390	E	A

### NA_N342D_ increased the viral pathogenicity in vivo

**Figure 5.**
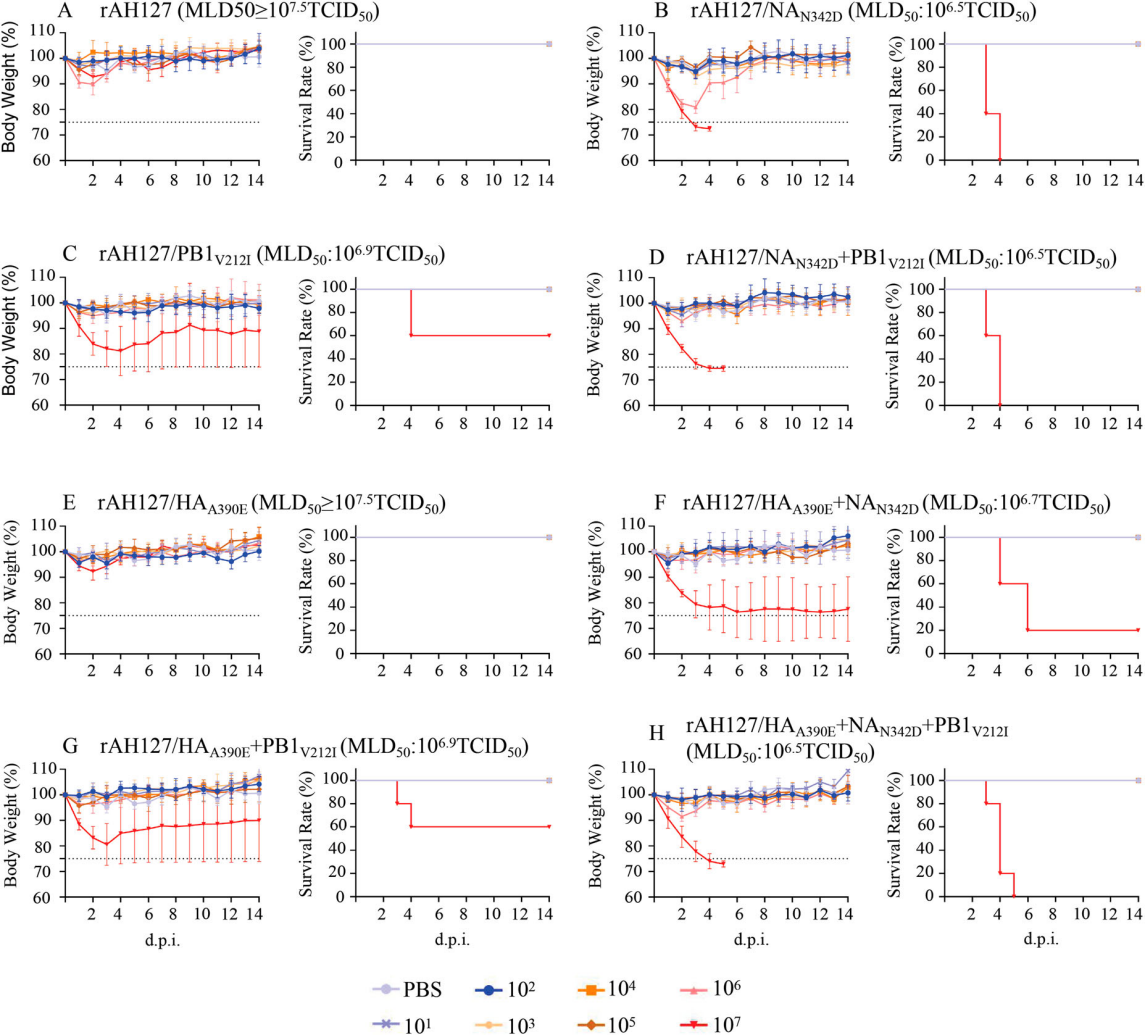
Virulence of the recombinant IBVs *in vivo.* (A–H) Five mice per group were inoculated intranasally with 10^1^ to 10^7^ TCID_50_ of rAH127 (A), rAH127/NA_N342D_ (B), rAH127/PB1_V212I_ (C), rAH127/NA_N342D_+PB1_V212I_ (D), rAH127/HA_A390E_ (E), rAH127/HA_A390E_+NA_N342D_ (F), rAH127/HA_A390E_+PB1_V212I_ (G), or rAH127/HA_A390E_+NA_N342D_+PB1_V212I_ (H). Body weight changes (left panels) and survival (right panels) of the mice were monitored daily. The error bars for body weight show the standard errors of the mean (SEM).

The impacts of the NA_N342D_ and PB1_V212I_ substitutions on the virulence of the wild type rAH127 were next investigated *in vivo*. As shown in Figure 5, 10^1^–10^7^ TCID_50_ doses of all recombinant rAH127 and rAH127/HA_A390E_ viruses were nonlethal to mice, each with a high MLD_50_ of more than 10^7.5^ TCID_50_ (Figure 5(A,E)). However, mice infected with 10^7^ TCID_50_ of the rAH127/NA_N342D_, rAH127/NA_N342D_+PB1_V212I_ or rAH127/HA_A390E_+ NA_N342D_+PB1_V212I_ showed a 100% fatality rate with an MLD_50_ value of 10^6.5^ TCID_50._ Mice infected with rAH127/HA_A390E_+NA_N342D_ exhibited a 20% survival rate and an MLD_50_ value of 10^6.7^ TCID_50_. Mice infected with rAH127/PB1_V212I_ or rAH127/HA_A390E_+PB1_V212I_ showed a 60% survival rate and an MLD_50_ value of at 10^6.9^ TCID_50_. HI antibody test results revealed showed that the MID_50_ values of rAH127/NA_N342D_, rAH127/PB1_V212I_ and rAH127/HA_A390E_ were lower than that of rAH127 (Table 1, Supplementary Table 2).

**Figure 6.**
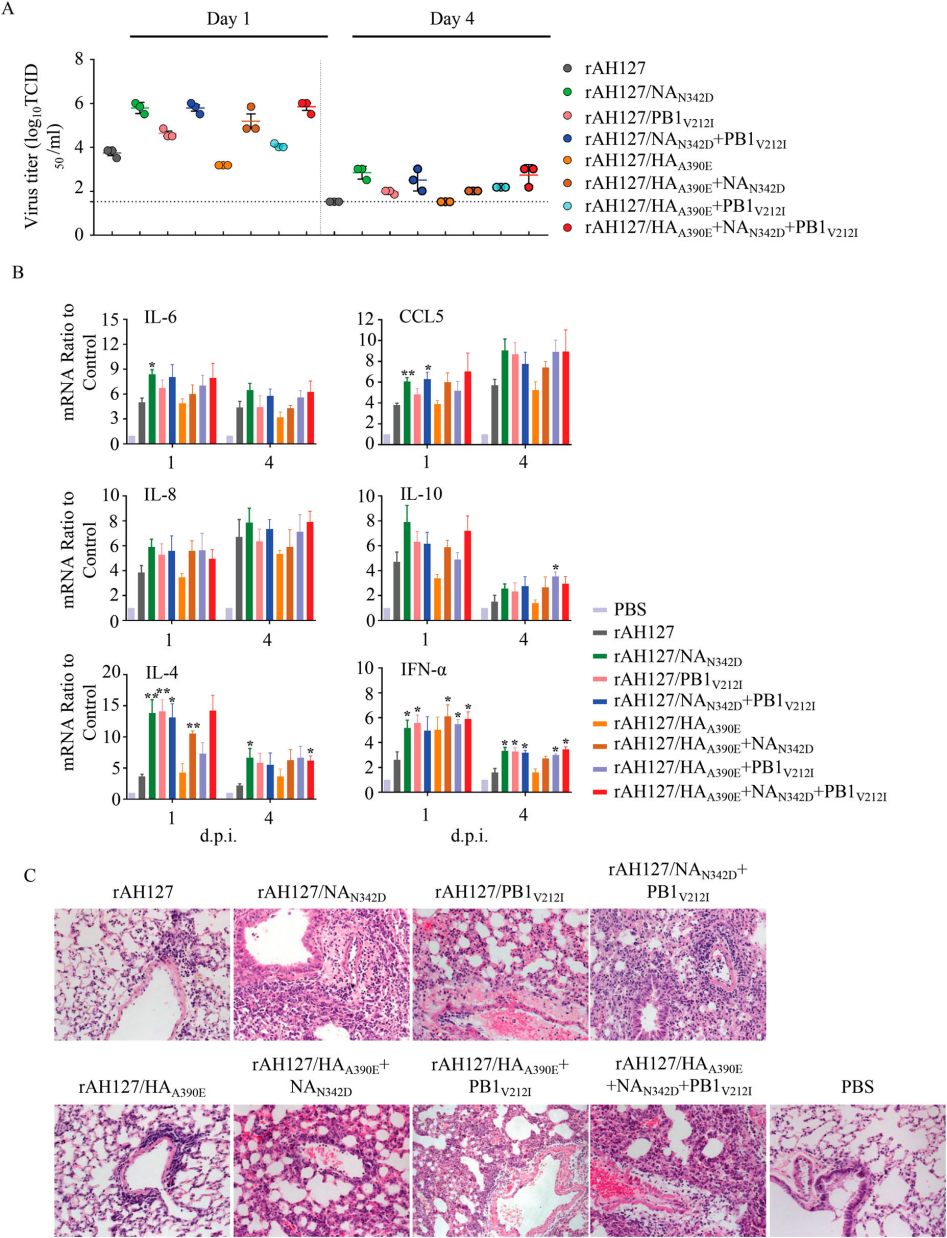
Viral replication and pathological changes in mice infected with the recombinant IBVs. Mice were infected with 10^7^ TCID_50_ of the recombinant IBVs. Three mice from each group were euthanized at 1 and 4 dpi. (A)The viral titres in mouse lungs were determined by performing TCID_50_ assays. (B) The mRNA levels of cytokines and chemokines in lung were determined by performing real-time PCR. (C) The histopathological lesions in the lungs, Scale bars, 100 µm. Statistical significance compared with mice infected with the rAH127 virus was determined by a *t*-test, *indicates *p* < 0.05, ** indicates *p* *<* 0.01.

In murine lungs, rAH127/HA_A390E_ replicated as well as the wild type rAH127. However, rAH127/PB1_V212I,_ and rAH127/NA_N342D_ replicated to significantly higher titres compared with rAH127 at both 1 and 4 d.p.i. (Figure 6(A)). Correspondingly, a histological analysis revealed that mice infected with either rAH127 or rAH127/HA_A390E_ displayed slight amounts of inflammatory infiltration. In contrast, mice infected with mutant viruses containing rAH127/NA_N342D_ and/or rAH127/PB1_V212I_ showed severe bronchopneumonia inflammation and inflammatory cell infiltration (Figure 6(C)). The expression levels of IL-4, IL-6, IL-8, IL-10, CCL5 and IFN-α in mouse lungs were 1–4-fold higher at 1 d.p.i and 1–3-fold higher at 4 d.p.i in tissues from mice infected with rAH127/NA_N342D_ or rAH127/PB1_V212I_ viruses infected mice compared with those from mice infected with rAH127 or rAH127/HA_A390E_ (Figure 6(B)). Overall, HA_A390E_ did not affect IBV replication and PB1_V212I_ induced slightly higher viral replication activity, whereas, NA_N342D_ significantly enhanced viral replication as well as pathogenicity both *in vitro* and *in vivo*.

### NA_N342D_ enhanced IBV neuraminidase activity

**Figure 7.**
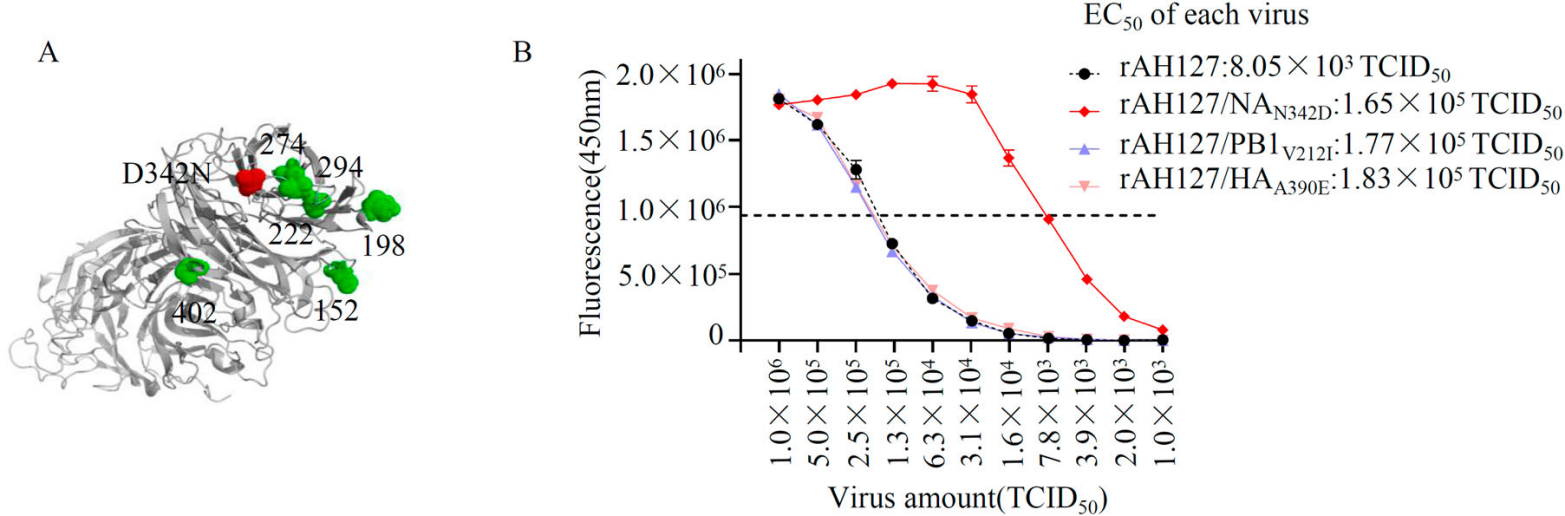
NA_N342D_ enhances IBV neuraminidase activity. (A) Known locations in or near the IBV neuraminidase active sites are highlighted in the modelled protein structure of the IBV NA. The N342D substitution is shown in red, previously described mutations are shown in green. (B) The NA activities of these viruses were detected in the presence of 200 µM MUNANA. The reactions were analysed at excitation and emission wavelengths of 365 nm and 450 nm, respectively. The dashed line shows the half-maximal effective concentration (EC_50_).

To investigate the molecular mechanism by which NA_N342D_ increased IBV virulence, we constructed 3D models of the tetrameric NA. As shown in Figure 7(A), position of 342 is located near the enzyme active sites at positions 274 and 294 in the NA structure [33,34], indicating that an N342D substitution might affect the neuraminidase activity. To assess this, the neuraminidase activity levels of the wild type rAH127 and single-mutant (rAH127/NA_N342D_, rAH127/PB1_V212I_, and rAH127/HA_A390E_) viruses were measured. Our data show that the neuraminidase enzyme activity of rAH127/NA_N342D_ was higher than that of rAH127, and the half-maximal effective concentration (EC_50_) value of rAH127/NA_N342D_ was ∼21-fold lower than that of rAH127, whereas those of rAH127/PB1_V212I_ and rAH127/HA_A390E_ were similar to that of rAH12. (Figure 7(B)). These results indicated that the NA_N342D_ mutation dramatically upregulated the neuraminidase activity of IBV.

## Discussion

IBV is one of the most important human respiratory pathogens that causes 20–30% of the total seasonal influenza burden every year [2]. However, unlike IAV, little is known about the molecular pathogenic determinants of IBV. The previous reported molecular determinants of IBVs were identified from lung-to-lung passages in mice [14–16].

In the present study, we found that two natural IBVs (AH1528/14 and AH127/15) exhibit divergent growth properties in cells and pathogenicity in mice (Figures 2 and 3). Despite these different phenotypes, only three amino acids differences were detected in the full genomes of these two viruses (Table 2). The subsequent *in vitro* and *in vivo* experiments revealed that these three substitutions provide different contributions to viral replication and pathogenicity. The HA_A390E_ was found to have no effect on the replication abilities of IBVs in cells or pathogenicity of IBVs in mice (Figure 4(C) and 5(E)). IBVs with a single PB1_212I_ substitution replicated with moderate efficiency in both MDCK cells and mice. An N342D substitution in the NA protein significantly enhanced the pathogenicity of IBVs in mice.

An A390E substitution located on the HA stalk region, might play a limited role in the viral replication and pathogenicity of IAV [35]. In contrast, we found here that HA_A390E_ did not affect either the viral replication or pathogenicity of IBV. PB1 is one of the viral polymerase proteins, which mediate viral transcription and replication [36]. Surprisingly, IBV containing PB1_212I_ exhibited a level of polymerase activity similar to that of IBV containing PB1_212V_ (Figure S2). Thus, the underlying mechanism for the PB1_V212I_ substitution needs to be studied further. Moreover, the enhanced pathogenicity contributed by the substitution N342D substitution in the NA protein may be due to the increased neuraminidase activity caused by this substitution (Figure 7(B)).

NA is a membrane-bound protein, and is located on the surface of influenza virus-infected cells and virions. It cleaves sialic acid from the cell surface during the final stages of the replication cycle, enabling the release of progeny virions [37]. For IAVs, an increased NA activity is correlated with the release of influenza viral RNA-containing particles into the air. Higher NA enzyme activity in the 2009 pandemic H1N1 viruses increased the release of virus particles and therefore improved virus transmissibility [38]. K110E mutation in NA significantly promoted NA enzyme activity and enhanced mice pathogenicity combined with PB2 mutations in H10N7 [39]. For IBV, a previous study found that a single substitution from D to N at position 457 of NA destroyed the salt bridge acting as a contact surface between the monomers. Thus, this change caused a significant loss in enzymatic activity but did not impact viral replication *in vitro* [40]. In our study, based on a 3D structure analysis, reside 342 in the NA protein is located closely to the enzyme active site (Figure 7(A)), and NA_N342D_ mutation did increase the neuraminidase activity (Figure 7(B)). It could be one of the causative factors for why NA_N342D_ could dramatically increased IBV virulence.

In conclusion, we found that NA_N342D_ could significantly enhance the viral infectivity, replication, and pathogenicity of IBVs. The identification of this novel pathogenicity determinant of IBVs could provide novel insight into future pathogenicity studies and surveillance of IBVs.
